# Dengue vaccine: local decisions, global consequences

**DOI:** 10.2471/BLT.15.168765

**Published:** 2016-09-07

**Authors:** Hugo López-Gatell, Celia M Alpuche-Aranda, José I Santos-Preciado, Mauricio Hernández-Ávila

**Affiliations:** a National Institute of Public Health, Avenida Universidad 655, Santa María Ahuacatitlán, Cuernavaca, 62100, Mexico.; b Division of Experimental Medicine, National Autonomous University of Mexico, Mexico City, Mexico.

## Abstract

As new vaccines against diseases that are prevalent in low- and middle-income countries gradually become available, national health authorities are presented with new regulatory and policy challenges. The use of CYD-TDV – a chimeric tetravalent, live-attenuated dengue vaccine – was recently approved in five countries. Although promising for public health, this vaccine has only partial and heterogeneous efficacy and may have substantial adverse effects. In trials, children who were aged 2–5 years when first given CYD-TDV were seven times more likely to be hospitalized for dengue, in the third year post-vaccination, than their counterparts in the control group. As it has not been clarified whether this adverse effect is only a function of age or is determined by dengue serostatus, doubts have been cast over the long-term safety of this vaccine in seronegative individuals of any age. Any deployment of the vaccine, which should be very cautious and only considered after a rigorous evaluation of the vaccine’s risk–benefit ratio in explicit national and subnational scenarios, needs to be followed by a long-term assessment of the vaccine’s effects. Furthermore, any implementation of dengue vaccines must not weaken the political and financial support of preventive measures that can simultaneously limit the impacts of dengue and several other mosquito-borne pathogens.

## Résumé

À mesure que de nouveaux vaccins contre des maladies très répandues dans les pays à revenu faible et intermédiaire deviennent disponibles, les autorités sanitaires nationales sont confrontées à de nouveaux défis règlementaires et politiques. L'utilisation du CYD-TDV, un vaccin vivant atténué, chimérique et tétravalent contre la dengue, a récemment été approuvée dans cinq pays. Bien qu'il soit prometteur pour la santé publique, ce vaccin n’a qu’une efficacité partielle et hétérogène et pourrait avoir d'importants effets indésirables. Dans les essais, les enfants âgés de 2 à 5 ans lors de la première administration du CYD-TDV avaient sept fois plus de risques d'être hospitalisés pour la dengue au cours de la troisième année après la vaccination que leurs homologues du groupe témoin. Comme il n'a pas été précisé si cet effet indésirable est uniquement lié à l'âge ou s'il est déterminé par le statut sérologique de la dengue, des doutes planent sur l'innocuité à long terme de ce vaccin chez les personnes séronégatives de tout âge. Tout déploiement de ce vaccin, qui devrait se faire de manière très prudente et réfléchie, après une évaluation rigoureuse du rapport bénéfices-risques dans des scénarios nationaux et sous-nationaux explicites, devrait être suivi par une évaluation à long terme de ses effets. En outre, la vaccination contre la dengue ne doit pas fragiliser le soutien politique et financier en faveur de mesures préventives, qui peuvent dans le même temps limiter l'impact de la dengue et de plusieurs autres pathogènes transmis par les moustiques.

## Resumen

A medida que nuevas vacunas contra enfermedades prevalentes en países con ingresos bajos y medios están cada vez más disponibles, las autoridades sanitarias nacionales se enfrentan a nuevos desafíos legislativos y políticos. Recientemente, cinco países han aprobado el uso de la CYD-TDV, una vacuna contra el dengue quimérica tetravalente de virus vivos atenuados. A pesar de ser prometedora para la salud pública, esta vacuna sólo tiene una eficacia parcial y heterogénea, y puede presentar efectos enormemente perjudiciales. En los ensayos, los niños de entre 2 y 5 años tratados con CYD-TDV por primera vez tuvieron una probabilidad de ser hospitalizados por el dengue, durante el tercer año tras la administración de la vacuna, siete veces mayor que sus homólogos del grupo de control. Dado que no se ha aclarado si este efecto perjudicial es únicamente cuestión de edad o si está determinado por el estado serológico del dengue, se ha puesto en duda la seguridad a largo plazo de esta vacuna en individuos seronegativos de cualquier edad. La vacuna, que debería administrarse con precaución y tenerse en consideración únicamente tras una evaluación rigurosa del coeficiente de riesgo y beneficio de la misma en escenarios nacionales y subnacionales concretos, debe ser objeto de seguimiento a largo plazo para evaluar sus efectos. Asimismo, la implementación de las vacunas contra el dengue no debe debilitar el apoyo político y financiero a medidas preventivas que puedan limitar los impactos del dengue y, al mismo tiempo, varios patógenos transmitidos por picaduras de mosquitos.

## ملخص

في الوقت الذي تتوفر فيه تدريجيًا لقاحات جديدة لمقاومة الأمراض المنتشرة في البلدان ذات الدخل المنخفض والمتوسط، تواجه الجهات الوطنية المسؤولة عن الناحية الصحية مزيدًا من التحديات الجديدة المتعلقة باللوائح التنظيمية والسياسات. وتمت الموافقة مؤخرًا على استخدام لقاح حمّى الضنك "CYD-TDV" – وهو لقاح خيمري رباعي التكافؤ مخفف وحي – في خمسة بلدان. وبالرغم من أنه لقاح واعد لتحسين الصحة العامة، فهو لا يقدم سوى مقدار جزئي ومتباين من الكفاءة، وقد يؤدي إلى حدوث آثار مناوئة وملموسة. في التجارب التحليلية، كان الأطفال الذين تتراوح أعمارهم من 2 إلى 5 أعوام عندما تلقوا لقاح CYD-TDV أول مرة تزيد أرجحية دخولهم إلى المستشفى بمعدل سبع مرات أكثر نتيجة إصابتهم بحمّى الضنك في العام الثالث بعد تلقيهم التطعيم عن قرنائهم من الأطفال في المجموعة الشاهدة. وبينما لم يتم توضيح ما إذا كان التأثير الخطير يعود فقط إلى عامل العمر أو أنه يتم تحديده من خلال الوضع المصلي لحمّى الضنك، فإن الشكوك قد أحاطت بمدى سلامة اللقاح على المدى البعيد بالنسبة لمجموعة الأفراد سلبية المصل بمختلف الأعمار. ينبغي أن تتم أي عملية لتوزيع ونشر اللقاح مع مراعاة الحذر الشديد، مع عدم اللجوء إليها إلا بعد إجراء تقييم مكثف لنسبة الخطورة والفائدة من تلقي اللقاح في ظل السيناريوهات الواضحة على الصعيد الوطني ودون الوطني، وعلى أن يتبعها إجراء تقييم طويل الأجل لآثار اللقاح. وعلاوة على ذلك، ينبغي ألا تكون عمليات توفير وتقديم لقاحات حمّى الضنك سببًا في إضعاف الدعم السياسي والمالي لإجراء التدابير الوقائية التي يمكنها الحد في الوقت نفسه من آثار حمّى الضنك والعديد من مسببات الأمراض الأخرى التي تنتقل عن طريق البعوض.

## 摘要

随着抵抗中低收入国家流行疾病的新疫苗逐渐面市，各国卫生当局正面临着新的法规和政策挑战。 CYD-TDV——一种四价嵌合登革热减毒活疫苗——最近获五个国家批准使用。 尽管在公共卫生领域前景光明，但此疫苗仅起到部分且不均匀的疗效，并且可能会造成严重不良反应。 经试验，与对照组同龄人相比，2-5 岁儿童在首次接种 CYD-TDV 后的第三年因登革热入院的可能性增加了七倍。 由于目前尚未确定该不良反应是仅由年龄机能造成，还是由登革热感染状况所导致，人们对该疫苗用于任何年龄段、血清呈阴性的个人的长期安全性产生了疑虑。 任何对该疫苗的使用均应非常谨慎，并且仅在具体国家和地区环境中通过严格的疫苗风险评估之后才进行考虑，随后需要对疫苗效果进行长期评估。 此外，对于可同时限制登革热和其它几种蚊虫传播病原体的预防措施，任何登革疫苗的使用均不得削弱对此类预防措施的政治和财政支持。

## Резюме

По мере того как новые вакцины для борьбы с заболеваниями, распространенными в странах с низким и средним уровнем дохода, становятся все более доступными, национальные органы здравоохранения сталкиваются с новыми проблемами законодательного и стратегического характера. Использование вакцины CYD-TDV — химерной тетравалентной вакцины с ослабленным возбудителем от лихорадки денге — было недавно одобрено в пяти странах. Хотя результаты применения вакцины и выглядят многообещающими для здравоохранения, она эффективна не во всех случаях и могут наблюдаться серьезные неблагоприятные явления. В клинических исследованиях было выявлено, что дети в возрасте от 2 до 5 лет, которых вакцинировали CYD-TDV, на третий год после вакцинации госпитализировались с лихорадкой денге в 7 раз чаще по сравнению со сверстниками из контрольной группы. Поскольку не было выяснено, связано это неблагоприятное явление только с возрастом детей или серостатусом по денге, была подвергнута сомнению безопасность данной вакцины в длительной перспективе для серонегативных лиц любого возраста. Начинать применять вакцину следует осторожно и только после строгой оценки соотношения риска и пользы на уровне национальных и субнациональных сценариев. Это должно сопровождаться обязательной оценкой долгосрочных эффектов. Кроме того, внедрение вакцин против лихорадки денге не должно ослаблять политическую и финансовую поддержку профилактических мероприятий, которые могли бы одновременно ограничивать влияние лихорадки денге и некоторых других заболеваний, передаваемых через укусы комаров.

## Introduction

Complex political, social and ecological factors drive dengue dynamics and hinder dengue control.^1^^,^^2^ A safe, effective and affordable dengue vaccine that is tetravalent – i.e. effective against all four serotypes of the dengue virus – has long been sought. CYD-TDV – a chimeric tetravalent, live-attenuated dengue vaccine – was developed by Sanofi Pasteur (Lyon, France) and recently licensed, for use in individuals aged 9–45 years, in five low- or middle-income countries: Brazil, Costa Rica, El Salvador, Mexico and Philippines.^3^

On 15 April 2016 the World Health Organization’s (WHO’s) Strategic Advisory Group of Experts on Immunization – hereafter called the advisory group – recommended that countries should consider implementation of CYD-TDV in national or subnational territories where at least 70% of the members of the age group targeted for vaccination are seropositive for dengue virus.^4^ The advisory group also discouraged the vaccine’s use in areas where less than 50% of the members of the targeted age group are seropositive.^4^ Before making these recommendations, the advisory group thoroughly analysed the published evidence of the vaccine’s safety and efficacy^5^ as well as relevant unpublished information that had been provided, on request, by Sanofi Pasteur. Although much of this analysis focused on the results of two large-scale, multicentre, Phase-III clinical trials,^6^^–^^8^ part of it was based on the comparative modelling of the potential public health impact of CYD-TDV’s deployment.^9^ The advisory group indicated that the vaccine should only be deployed as one of a set of dengue control measures that also included functional programmes of vector control and robust surveillance. The advisory group left it to individual countries to assess whether their local priorities reasonably justified the deployment of CYD-TDV.

Resolutions made by national regulatory authorities on licensing new vaccines – and by national health authorities on implementing such vaccines – can influence the global regulatory framework for vaccines and the global systems of vaccine delivery. The recent licensing of CYD-TDV has challenged the capacity of countries to decide if, when and how they should deploy this vaccine and whether they have – or can soon develop – the capability to monitor the vaccine’s performance in the field. The latter capability appears essential given current concerns – discussed below – over the vaccine’s partial efficacy and long-term safety and the duration of the immunity that it creates.

We worry that the licensing of CYD-TDV in one country may encourage the too rapid licensing of the vaccine in other countries that have weaker regulatory capacity. Although the related recommendations of the advisory group are valuable, they may be based on the optimistic assumption that all of the countries where CYD-TDV might be deployed are able to assess the risks and benefits of such deployment adequately and to respond well to any adverse effects – including those that only become apparent in the long term.

## Efficacy of CYD-TDV

Although the results from randomized clinical trials have illustrated the merits of CYD-TDV, they have also revealed the vaccine’s partial and heterogeneous efficacy in the prevention of dengue disease.^5^ Analyses of the trials’ pooled data indicated that, within 2 years of the first injection, the vaccine was moderately efficacious (mean: 60.3%; 95% confidence interval, CI: 55.7–64.5%) at protecting against symptomatic virologically confirmed dengue. However, efficacy varied substantially – and sometimes fell to zero – according to the vaccinated individual’s age and dengue serostatus at the time of vaccination and the infecting dengue serotype. Among individuals who were aged 9–16 years when first vaccinated, the estimated pooled efficacies of the vaccine were 81.9% (95% CI: 67.2–90.0%) and 52.5% (95% CI: 5.9–76.1%), respectively, for those who were seropositive for dengue and those who were seronegative – i.e. dengue-naïve – when first immunized. The corresponding efficacies for children who were younger than 9 years when first vaccinated were lower: 70.1% (95% CI: 32.3–87.3%) and 14.4% (95% CI: −111.0–63.5%), respectively. These findings indicate that CYD-TDV may act only as a booster of natural immunity and not as a vaccine to prevent primary dengue infection.^5^ No study has assessed directly the protective efficacy of the vaccine against virologically confirmed dengue in individuals aged more than 16 years.

Vaccine efficacy against symptomatic virologically confirmed dengue also seemed to vary according to the serotype of the infecting dengue virus, with lower levels of protection against serotypes 1 (54.7%; 95% CI: 45.4–62.3%) and 2 (43.0%; 95% CI: 29.4–53.9%) than against serotypes 3 (71.6%; 95% CI: 63.0–78.3%) and 4 (76.9%; 95% CI: 69.5–82.6%).^8^ Similar age-related and serotype-related patterns were observed in the pooled estimated efficacies against severe dengue.^8^

## Safety of CYD-TDV

During the initial 2 years of active surveillance in the randomized Phase III trials of CYD-TDV, acute adverse events were similarly infrequent in the vaccine and control groups. However, extended hospital-based observation in the third year of surveillance – i.e. year 3 – revealed that, in Asia, the vaccine was associated with a relative risk of hospitalization, for virologically confirmed dengue, of 1.58 (95% CI: 0.83–3.02) in children who were younger than  9 years when first vaccinated and 7.45 (95% CI: 1.15–314) in children who were aged 2–5 years when first vaccinated.^8^ Likewise, the estimated relative risk of severe dengue during follow-up years 3 to 5 was 6.47 (95% CI: 0.97–275) in children who were younger than  9 years when first vaccinated and 3.53 (95% CI: 0.45–159) in children who were aged 9–11 years when first vaccinated.^5^ Although some of these differences are not statistically significant, they have raised concerns over the long-term safety of CYD-TDV, particularly among individuals who are seronegative when first vaccinated.^10^^–^^12^

In the clinical trials of CYD-TDV conducted so far, the numbers of seronegative subjects have been too small and the follow-up periods have been too short to reach any firm conclusion regarding the safety of the vaccine when used on dengue-naïve individuals of any age.^5^ Some of the available data do, however, indicate that the potential of this vaccine to increase the risk of severe dengue and other forms of dengue that lead to hospitalization^13^^,^^14^ is more than theoretical. It seems possible that exposure to CYD-TDV predisposes the dengue-naïve to a secondary-like dengue infection when they are first exposed to dengue virus.^15^^–^^17^ The comparative modelling of the public health impact of CYD-TDV was based on this hypothesis.^9^ However, researchers at Sanofi Pasteur have offered two other explanations: (i) that, because of their immature vascular systems, young children may be particularly susceptible to – and particularly slow to recover from – severe dengue, and (ii) that deployment of the vaccine accelerates the clustering of susceptibility to dengue in a population.^5^^,^^18^

## National licensing

A central tenet of the Dengue Vaccine Initiative has been to help narrow the gap between the development of dengue vaccines and access to such vaccines in areas where dengue is common.^19^ Although facilitating access to dengue vaccines in those countries that are worst afflicted by dengue is a noble goal, the early adoption of any new vaccine in any country should be limited by the capacity of that country’s national health and regulatory authorities to appraise the evidence for that vaccine’s cost–effectiveness ratio, efficacy, local relevance and safety and the likelihood that the vaccine’s deployment will improve public health. The hasty deployment of a new vaccine – before the associated risks and benefits can be carefully evaluated in existing, real-life settings – should be avoided.

Although Mexico was the first country to approve CYD-TDV,^5^ the results of the clinical trials in five countries in Latin America – i.e. Brazil, Colombia, Honduras, Mexico and Puerto Rico – and five in Asia – i.e. Indonesia, Malaysia, the Philippines, Thailand and Viet Nam – indicated that the vaccine was markedly less efficacious in Mexico than in any of the other nine countries. In Mexico, a relatively small proportion (53%) of the trial participants – who were recruited in areas endemic for dengue – were seropositive and almost all (95%) of the dengue infections were attributed to serotypes 1 or 2.^7^

Most vaccines used in low- and middle-income countries were deployed in those countries only after they had been licensed and widely used in high-income countries. The populations of low- and middle-income countries have therefore been protected, against poor vaccines, by the results of careful post-marketing surveillance by strong regulatory agencies such as the European Medicines Agency and the United States Food and Drug Administration. In several low- and middle-income countries, national regulatory authorities are now considering the deployment of CYD-TDV – a vaccine that has only been assessed in clinical trials^20^ – even though they may be relatively weak and relatively susceptible to the pharmaceutical industry’s influence and have not set explicit criteria for assessing vaccine efficacy or safety.

## Post-licensing surveillance

In the few countries whose national regulatory authorities have approved CYD-TDV, evaluation of the vaccine’s long-term safety and efficacy has now been deferred to post-licensing surveillance. The follow-up for the ongoing CYD-TDV clinical trials, which is expected to end between November 2017 and April 2018, has not yet run for the minimum period, of three to five years, recommended by WHO.^21^

Other countries planning to implement CYD-TDV-based vaccinations must also be prepared and able to evaluate the vaccine’s long-term safety. Any post-licensing surveillance must be sufficient to determine if the vaccine’s benefits outweigh its adverse effects – especially the risk of hospitalization for dengue or severe dengue, among individuals who are dengue-naïve when first vaccinated.^22^ The results from a few clinical trials should not be used to predict the vaccine’s value when used in populations with varying dengue seroprevalence, varying levels of vector control and clinical care and dissimilar distributions of the other predictors of vaccine uptake and response.^23^ Although, in previous modelling, several apparently realistic scenarios were explored,^9^ the assumptions that had to be made may have weakened the representation of national and subnational heterogeneity.

CYD-TDV, like many other vaccines, is selectively efficacious but, unusually, its safety apparently depends on the prevalence of the target disease in the population being vaccinated. The most benign potential consequence is that the population effectiveness of the vaccine will wane as the prevalence of dengue declines over time.^24^ Depending on dengue serostatus at the time of vaccination, CYD-TDV may protect, be a wasteful intervention or harm the vaccinees.^5^ Ideally, to reduce risks, the pre-vaccination dengue serostatus of each vaccine target should be determined. However, as no point-of-care rapid test for dengue infection is yet available, it has been suggested that seroprevalence be evaluated at population level – i.e. as an indicator of probable dengue exposure at the level of the individual. In many low-income countries, however, even the evaluation of seroprevalence at population level may put too much strain on public health infrastructures.

The advisory group has recommended that countries consider CYD-TDV vaccination where dengue seroprevalence is at least 70% and refrain from deploying the vaccine where such seroprevalence is lower than 50%.^4^ The advisory group left it unclear what countries should do with the vaccine in areas where dengue seroprevalence lies between 50% and 69%. The transmission of dengue virus is temporally and geographically heterogeneous and several environmental, social and behavioural variables determine people’s exposure to the virus. Consequently, the dengue seroprevalence in one community may lie below 50% while that in a neighbouring community – within a country or even within a province or a city – may lie above 69%.^5^ To save money, most countries conducting pre-vaccination serosurveys of dengue would prefer to use parsimonious sampling schemes that will fail to detect such small-scale heterogeneity. If we are to optimize the risk–benefit ratio for the deployment of CYD-TDV, we need global standards for serosurvey design, quality assurance and control, laboratory methods and data analysis.

The results of pre-vaccination serosurveys may represent the target population’s history of natural exposure to dengue virus. However, once vaccinations have begun, new cohorts of dengue-naïve and dengue-exposed individuals will mix with individuals who have become seropositive for dengue as the result of vaccination. The results of any follow-up serosurveys will represent a mix of vaccine-derived immunity and natural immunity and it remains unclear if these two types of immunity differ in their biological and epidemiological significance.

Any post-licensing surveillance must be able to detect and assess any augmented risk of dengue disease that requires hospitalization – which may be reflected as a post-vaccination increment in the mean clinical severity of dengue cases – and any waning in the protective efficacy of CYD-TDV over time. Conventional post-licensing surveillance, as seen in countries with established pharmacovigilance systems, generally focuses on adverse effects that are clinically distinct from the signs and symptoms of the target disease and that occur within a few weeks of the vaccinations. Such surveillance could easily miss the adverse effects of CYD-TDV, which may be indistinguishable from dengue disease and – as already seen in clinical trials – take years to appear. With CYD-TDV, it may be particularly difficult to distinguish between vaccine failure and vaccine-induced disease. Although Sanofi Pasteur has proposed a post-licensing risk management plan that includes event monitoring with a cohort of vaccine recipients,^5^ this approach is weakened by the absence of credible reference values. The thorough monitoring of unvaccinated seropositive and seronegative controls will be crucial in evaluating the possible association between vaccination and the risk of severe disease.^14^^,^^25^

Safety monitoring of CYD-TDV must be long enough for natural exposure to dengue virus to occur and the study populations must be large enough to allow sufficient statistical power to estimate the relative risks of all of the relevant outcomes – according to vaccination and baseline dengue serostatus.^21^

Many low- and middle-income countries recognize the need to improve their vaccine safety systems. Only a few currently have the comprehensive pharmacovigilance and health surveillance systems needed to monitor, report and evaluate complex safety issues – such as those associated, at least potentially, with CYD-TDV.^24^ In its recent evaluation of the Mexican National Immunization Programme, the Pan American Health Organization found the system for the reporting of vaccine-related adverse events to be one of the programme’s key components that needed strengthening.^26^

It remains unclear which organization, independent from industry, will compile and analyse information regarding CYD-TDV-related outcomes systematically and assure global monitoring of the probably sparse data collected in countries where this vaccine may be used. The reference values used to guide the assessment of the vaccine’s risk–benefit ratio – and the risk thresholds that have to be crossed to trigger any public health intervention – still have to be set. It is also unclear if countries are prepared to respond to any substantial adverse effects – detected at global, regional or national level – in a timely fashion.

## Global consequences

Although resolutions on the licensing and eventual deployment of CYD-TDV are local, they may affect the global regulatory framework and global programmes of dengue control in at least five ways. First, the deployment of CYD-TDV poses novel logistical and administrative challenges. For example, although most countries set nationwide immunization schedules – to simplify operations and reduce costs – the apparent association between CYD-TDV’s efficacy and pre-vaccination serostatus will force schedules to be set on smaller geographical scales, complicate vaccine delivery and increase costs. Second, health authorities will have to explain to the public why some communities are immunized while others, with seemingly similar exposure to dengue, are excluded. There is a possibility that CYD-TDV’s deployment will provoke so much public concern and public and health worker confusion that its deployment and other vaccination campaigns are weakened. Third, an unsubstantiated perception that an effective vaccine against dengue is available may discourage political and financial commitment to vector control, effective surveillance and other important preventive measures that remain necessary to confront dengue and other mosquito-borne diseases.^27^ The recent emergence of two other *Aedes*-borne arboviruses in the Americas, chikungunya and Zika, is a timely reminder of the continued importance of mosquito control. The advisory group was clear in recommending the vaccine only as a component of well established public health programmes – although it failed to stipulate a minimum level of performance, for vector control, clinical care and surveillance systems that would justify the vaccine’s deployment. Fourth, for CYD-TDV, the balance between expected benefits and identifiable hazards is complex. In particular, the vaccine appears to offer no clear benefit for dengue-naïve individuals who, if the vaccine does increase their risk of subsequent hospitalization, should probably avoid the vaccine. Attempts to communicate this dilemma to the public may discourage vaccine uptake while concealing this information could severely damage public trust. Fifth, WHO established the Vaccines Prequalification Programme, 25 years ago, to assure the safety and effectiveness of vaccines used in the national immunization programmes of low- and middle-income countries. As this programme evolved, reliance on national regulatory authorities became the cornerstone of a trust-based system that protects the quality, safety and efficacy of vaccines procured by United Nations agencies.^28^ Recognizing that solid regulatory capacity is crucial to assure the global supply of safe vaccines, WHO is determined to strengthen the global regulatory framework for vaccines.^29^ Authorization of an unsafe vaccine by a WHO-recognized regulatory agency would damage the confidence that supports the Vaccines Prequalification Programme and global vaccine supply.

The impact of dengue on public health may push endemic countries towards the rapid adoption of the first available dengue vaccine: CYD-TDV. Such a choice may be premature, however, given the limited and selective efficacy of CYD-TDV and the lingering uncertainty regarding its safety. In vaccine development, the relevant regulatory authorities must have access to all evidence that allows the potential adverse effects of a vaccine candidate to be evaluated and weighed carefully against the potential benefits.^30^ Local and global capacity for assessing the long-term safety of CYD-TDV in post-licensing surveillance must be strengthened to meet the challenges imposed by the vaccine’s complex performance. Before they deploy any dengue vaccine, countries must uphold their commitment to integrated and sustainable vector control, high-quality clinical care and robust surveillance.
